# Book Notices

**Published:** 1881-05-20

**Authors:** 


### *BOOK NOTICES.*

HYDROPHOBIA: A Monograph for the Profession and the Public. By Horatio R. Bigalow, M. D. Published by D. G. Brinton, M. D., 115 S. 7th St., Philadelphia. 1 Vol. 8vo. Cloth, pp. 154. Price $1.00.

This volume is designed for the Physician, the Veterinarian, and the intelligent general reader.

It treats in a thorough and exhaustive manner of that terrible disease *Hydrophobia*—the most terrible of any known to science. It relates its history, symptoms, preventive treatment, and the precautions to be taken against it, both public ones and those for single cases, and individual instances.

The author is a distinguished physician and a writer of celebrity. His researches have been most extensive, and he presents them in an easy and clear style. The questions of the management of such cases and the possible chances of recovery are fully discussed.

JOHN HUNTER AND HIS PUPILS: by S. D. Gross, M. D., L. L. D., Oxon., L. L. D., Cantab, Prof. of Surgery in the Jefferson Medical College; President of the Philadelphia Academy of Surgery, etc. Philadelphia, Pressley Blakiston, 1012 Walnut St. J. J. & S. P. Richards, Atlanta, Ga. Price $1.50.

This little work of 103 pages oc., containing an interesting sketch of the life, character and services of the renowned John Hunter, may be read with pleasure and instruction, by both the student and practitioner of medicine, everywhere.

WHAT EVERY MOTHER SHOULD KNOW: by Edward Ellis, M. D., late Senior Physician to the Victoria Hospital for women and children, and to the North London Hospital for consumption, etc. Author of a Practical Manual on the diseases of children, etc., Philadelphia. Pressley Blakiston, 1012 Walnut St., 1881. J. J. & S. P. Richards, Atlanta, Ga. Price 75 cts.

This is a very practical little work of 132 pages. It is the work of an eminent and able practitioner, and though plain enough to be comprehended by the unprofessional reader, is yet replete with useful matter which students and physicians throughout the country would do well to possess themselves of.

A GUIDE TO THE CLINICAL EXAMINATION OF PATIENTS AND THE DIAGNOSIS OF DISEASES: by Richard Hogan, M. D., Privat, docent to the University of Leipsic. Translated from the London revised and enlarged edition, by G. E. Gramm, M. D. Boericke & Tafel, New York.

This work is eminently practical.

A TREATISE—ALBUMINURIA: by W. Howship Dickenson, M. D., Contab. Fellow of the Royal College of Physicians; Physician to St. George Hospital—Sen'r Physician to the hospital for sick children—Corresponding Member of the Academy of Medicine, New York. Second edition. Wm. Wood & Co., New York, 1881. Oc. 300 p.

The above is one of Wood's Library Standard of Medical Authors. As it relates to a subject which is very imperfectly understood by the larger portion of the profession, it should be read and carefully studied by every practitioner. It is well gotten up and beautifully illustrated.

